# The complete mitochondrial genome of a planarian flatworm *Girardia tigrina* (Tricladida: Dugesiidae)

**DOI:** 10.1080/23802359.2021.1962757

**Published:** 2021-08-12

**Authors:** Xiu-Zhen Cheng, Dan Jin, Li-Li Duan, Wen-Song Xu, Hong-Chun Pan

**Affiliations:** College of Life sciences, Anhui Normal University, Wuhu, China

**Keywords:** *Girardia tigrina*, complete mitochondrial genome, Dugesiidae, phylogenetic analysis

## Abstract

*Girardia tigrina*, a freshwater planarian species native to America and introduced to other continents, has been usually used as model organism in many research fields of biology. In this study, we determined the complete mitochondrial genome of *G. tigrina* using next-generation sequencing (NGS). The complete mitogenome was 15,938 bp in length, with 36 genes, including 12 protein-coding genes, 2 ribosomal RNAs and 22 transfer RNAs, and ATP8 was absent in the mitogenome of *G. tigrina* as in the mitogenomes of some other flatworms. The maximum-likelihood phylogenetic tree suggested that *G. tigrina* was closely related to genus *Dugesia* in the clade of Tricladida.

*Girardia tigrina* is a freshwater planarian species native to America, and has been introduced to other continents such as East Asia and Europe. Due to its large body size (approximately 10 mm in length), fast asexual reproductive rate and easy large-scale cultivation in the laboratory, *G. tigrina* has become an important model organism in many research fields such as development, stem cell, regeneration, neurogenesis and toxicology (Lopes et al. 2015; Bach et al. 2016). In this study, we reported the complete mitochondrial genome of *G. tigrina* and relevant phylogenetic analysis for the first time (GenBank accession No: MW972220). The sample of *G. tigrina* was collected from Wuhu (31.32°N, 118.47°E), Anhui Province, China, in May 2020, and the specimen was deposited at the Nature Museum affiliated with Anhui Normal University (https://biology.ahnu.edu.cn, Hong-Chun Pan, panhongchun@126.com) under the voucher number GT202005p03. DNA materials were extracted using Sangon DNA sample preparation kit (Sangon, China), and genome sequencing was performed using the Illumina HiSeq2000 at Personalbio Biotechnologies Inc., Nanjing, China. Approximately 4.8 GB of clean data were obtained, then the trimmed reads were assembled by Geneious v. 8.1.9 (Kearse et al. 2012).

The complete mitogenome of *G. tigrina* was circular in shape and 15,938 bp in length with 27.62% GC content. In total, the mitogenome contained 36 genes that included 12 of the 13 protein-coding genes characteristic of metazoan mitogenomes, 2 ribosomal RNA (rRNA) genes, 22 tRNA genes, and a non-coding region (D-loop) of 845 nucleotides in length. Notably, ATP8 was absent in the mitogenome of *G. tigrina* as usually found in some other flatworms (Sakai and Sakaizumi 2012; Aguado et al. 2016). In addition, the nucleotide composition of the mitogenome was highly A + T biased, the A + T content of PCGs, tRNAs, and rRNAs was 72.42%, 70.26%, and 73.58%, respectively.

Phylogenetic analysis was based on 12 PCGs sequence of *G. tigrina* with the other 12 free-living flatworms, and one cnidarian species (*Sarcophyton trocheliophorum*) was chosen as outgroup. The phylogenetic tree was reconstructed by using the Maximum-likelihood (ML) method with 1000 bootstrap replicates through MEGA X software (Kumar et al. 2018). The result showed that *G. tigrina* was closely related to genus *Dugesia* in the clade of Tricladida, and the phylogenetic relationship among these free-living flatworms was ((((Tricladida) + Macrostomida) + (Polycladida)) + Catenulida) (Figure 1). In conclusion, the complete mitogenome of *G. tigrina* reported in this study provided useful information for the phylogeny and evolution analysis of Platyhelminthes.

**Figure 1.**
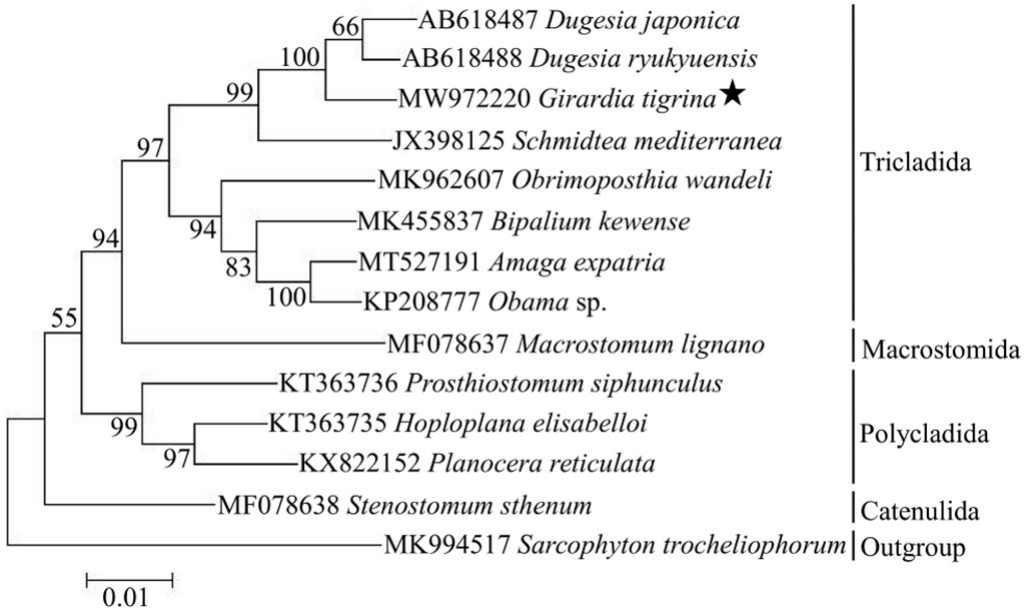
Maximum-likelihood tree based on 12 PCGs of 13 free-living flatworms by using MEGA X (bootstrap values based on 1000 replicates).

## Data Availability

The data that support the findings of this study are openly available in NCBI at https://www.ncbi.nlm.nih.gov/, reference number MW972220, PRJNA732802, SRR14654957, and SAMN19341100.
